# Development and validation of quick Acute Kidney Injury-score (q-AKI) to predict acute kidney injury at admission to a multidisciplinary intensive care unit

**DOI:** 10.1371/journal.pone.0217424

**Published:** 2019-06-20

**Authors:** Fiorenza Ferrari, Mariangela Valentina Puci, Ottavia Eleonora Ferraro, Gregorio Romero-González, Faeq Husain-Syed, Lilia Rizo-Topete, Mara Senzolo, Anna Lorenzin, Eva Muraro, Antonio Baracca, Mara Serrano-Soto, Alejandra Molano Triviño, Ana Coutinho Castro, Massimo De Cal, Valentina Corradi, Alessandra Brendolan, Marta Scarpa, Maria Rosa Carta, Davide Giavarina, Raffaele Bonato, Giorgio Antonio Iotti, Claudio Ronco

**Affiliations:** 1 International Renal Research Institute of Vicenza (IRRIV) and Department of Nephrology, Dialysis and Transplantation, San Bortolo Hospital, Viale Rodolfi, Vicenza, Italy; 2 Intensive Care Unit, San Bortolo Hospital, Viale Rodolfi, Vicenza, Italy; 3 Intensive Care Unit, I.R.C.C.S. Policlinico San Matteo, Viale Golgi, Pavia, Italy; 4 Department of Public Health, Experimental and Forensic Medicine, Unit of Biostatistics and Clinical Epidemiology, University of Pavia - Via Forlanini, Pavia, Italy; 5 RTS SAS, Medellín, Colombia; 6 Department of Internal Medicine II, Division of Pulmonology, Nephrology and Critical Care Medicine, University Clinic Giessen and Marburg - Campus Giessen, Giessen, Germany; 7 University Hospital “José Eleuterio González”, Francisco I Madero s/n and Gonzalitos Colonia Mitras Centro, Monterrey, Nuevo León, México; 8 DISCOG - Department of Surgery, Kidney and Pancreas Transplant, University of Padova, Padova, Italy; 9 abcGo.S.r.l. Viale Merello, Cagliari, Italy; 10 Nephrology Service Nephrology Service Marques de Valdecilla – Universitary Hospital, Santander, Cantabria, Spain; 11 Nephrology and Dialysis Service RTS - Fundación Cardioinfantil, Colombia; 12 Department of Nephrology, Dialysis and Transplantation, Oporto Hospital Center Largo Prof. Abel Salazar, Porto, Portugal; 13 Department of Laboratory Medicine, San Bortolo Hospital, Vicenza, Italy; Bambino Gesù Children’s Hospital, ITALY

## Abstract

AKI is associated with increased risk of death, prolonged length of stay and development of de-novo chronic kidney disease. The aim of our study is the development and validation of prediction models to identify the risk of AKI in ICU patients up to 7 days. We retrospectively recruited 692 consecutive patients admitted to the ICU at San Bortolo Hospital (Vicenza, Italy) from 1 June 2016 to 31 March 2017: 455 patients were treated as the derivation group and 237 as the validation group. Candidate variables were selected based on a literature review and expert opinion. Admission eGFR< 90 ml/min /1.73 mq (OR 2.78; 95% CI 1.78–4.35; p<0.001); SOFAcv ≥ 2 (OR 2.23; 95% CI 1.48–3.37; p<0.001); lactate ≥ 2 mmol/L (OR 1.81; 95% CI 1.19–2.74; p = 0.005) and (TIMP-2)•(IGFBP7) ≥ 0.3 (OR 1.65; 95% CI 1.08–2.52; p = 0.019) were significantly associated with AKI. For the q-AKI score, we stratified patients into different AKI Risk score levels: 0–2; 3–4; 5–6; 7–8 and 9–10. In both cohorts, we observed that the proportion of AKI patients was higher in the higher score levels.

## Introduction

Acute Kidney Injury (AKI) occurs in approximately 50% of patients admitted to an Intensive Care Unit (ICU). Increasing severity of AKI is associated with increased risk of death, prolonged length of stay, increased Intensive Therapy Unit utilisation, and the development of de-novo chronic kidney disease [1–5].

Currently, more than 200 different definitions of AKI are recorded in the literature worldwide [6]. In March 2012 the “KDIGO acute kidney injury clinical practice guidelines” [7] redefined RIFLE and AKIN criteria, and subsequent studies showed a better prediction performance of KDIGO compared to AKIN or RIFLE classifications in critically ill patients [8–12].

Due to kinetics, a significant rise of serum creatinine (SCr) or a reduction in urinary output (UO) occur 48–72 hrs after a kidney injury, and factors such as hydration, nutrition and lean tissue status further confound the diagnosis [7, 13]. Therefore, imprecise early identification of AKI depends on the definition itself of AKI, which is based on an increase in SCr or a decline in UO, both late and non-specific markers [7,13].

Furthermore, a grey-zone exists, as stage 0/A of Acute Kidney Disease (AKD), when no apparent residual injury is present, but the kidney might be vulnerable for some time after an episode of AKI [14]. AKI is a risk factor for the future loss of kidney function: the delay of approximately 24–48 h in elevating creatinine after AKI could promote iatrogenic injuries or lack of the monitoring of the renal function.

The primary goal for dealing effectively with AKI is to recognize its onset early to allow for timely appropriate interventions.

The aim of our study is the development and validation of prediction models to identify the risk of AKI in ICU patients up to 7 days.

## Material and methods

### Study design, setting and study population

This is a retrospective analysis of the (TIMP-2)•(IGFBP7) Vicenza registry. This registry has been enrolling consecutive critically ill patients admitted to the multidisciplinary ICU at San Bortolo Hospital since 1 June 2016. The registry inclusion criteria are: patients admitted to ICU who were over 18 and were fitted with a urinary catheter for at least 48 hrs, (TIMP-2)•(IGFBP7) ICU admission measurement, whereas the exclusion criteria are: advanced (stage 5) chronic kidney disease (CKD) [15], patients in anuria or with diuresis less than 30 ml within 24 hrs from ICU admission.

Study approval was obtained from the local Human Research Ethics Committee of the San Bortolo Hospital in Vicenza (protocol number 03/17), and the study complied with the Declaration of Helsinki. Informed consent was obtained under Italian laws (S1 File).

We recruited 692 consecutive patients admitted to ICU from 1 June 2016 to 31 March 2017: 455 patients were treated as the determination group and 237 as the validation group. A flow chart of the study population selection and research process is shown in S1 Fig.

AKI was staged each ICU stay day using the ‘Kidney Disease: Improving Global Outcomes’ (KDIGO) SCr criteria and urine output criteria [7]. For this retrospective study, to assess the baseline creatinine, we used the pre-morbid SCr measured 90–180 days before ICU admission [14].

### Laboratory data

Blood and urine samples were immediately collected at ICU admission. Urinary (TIMP-2)•(IGFBP7) was analysed with the Nephocheck Test (Astute Medical, San Diego CA, USA). All values for [TIMP-2]*[IGFBP7] are reported in units of (ng/ml)^2^/1000. Serum creatinine was measured by the enzymatic method (IL testTM, Instrumentation Laboratory SpA, Milano, Italy) on an ILab650 analyser (Instrumentation Laboratory, Werfen Group, Barcelona, Spain). All laboratory data were analysed by technicians who were blinded to the clinical data.

### Data collection

Patient data, demographic characteristics, body weight, height, body mass index (BMI). Comorbidities recorder from baseline included hypertension and diabetes mellitus along with previous use of insulin. Data collected at the time of ICU admission included the main reason for admission, severity of illness using the Simplified Acute Physiology Score II (SAPS II) and Sequential Organ Failure Assessment (SOFA), Mean Arterial Pressure (MAP), and in those patients with use of mechanical ventilation (MV), the Positive End-Expiratory Pressure (PEEP) and PaO2/FiO2 (PF) ratio were collected. Laboratory parameters measured included blood gas analysis, lactate and haemoglobin, as well as, within 24 hours of ICU admission, urine output (UO), cumulative fluid balance (CFB), maximum diuretic dose, higher vasopressors/inotropic drug dose(s), cardiovascular SOFA score, PEEP, Partial Pressure arterial Oxigen (PaO2) and Oxigen Inspired Fraction of Inspired Oxigen (FiO2) ratio (PaO2/FiO2), volume of concentrated red blood cell concentrated, and platelets transfused, along with blood gas analysis, lactate, haemoglobin and procalcitonin.

All Registry data was collected from the electronic health records (Digistat) of the ICU and the clinical laboratory using an Excel-based tool. The export of records for further processing and analysis has been anonymised.

Candidate variables and their cut-off were selected based on the literature review, expert opinion (an intensivist, two nephrologists) and availability in the dataset. The final set of predictor variables was determined incrementally for each increasingly complex model via bootstrapped backwards elimination analysis. S2 File shows the anonymized dataset of the study.

### Statistical analysis

To describe the sample, we used summary statistics expressed as means, standard deviations, median and IQR for continuous variables or percentages for qualitative ones. Normality distribution was assessed using the Shapiro-Wilk test. The ICU database sample was randomly split into two cohorts: derivation (66%; n = 455) and validation (34%; n = 237) cohorts.

To test the homogeneity of two groups we used T-test and chi square test or non-parametric equivalent test. Any variable with significant univariate test or clinical relevance was selected as a candidate for multivariate analysis.

After performing a multivariable logistic regression on derivation cohorts, stepwise forward process was used to select predictor variables. We subsequently checked for possible interactions and collinearity among predictors. The Hosmer-Lemeshow goodness of fit statistic was used on the best model, determined using both clinical and statistical criteria, to test the calibration. Discrimination performance of risk index was assessed using the area under the ROC curve, and the best cut-off point that maximized both sensitivity (Se) and specificity (Sp) was chosen. The integrated discrimination improvement (IDI) was reported.

Regression coefficients were used to derive an integer score for the development of an easy-to-use AKI risk score. Afterwards, the final AKI risk score model was assessed in the validation cohort using the area under the ROC curve and the Hosmer-Lemeshow goodness of fit test.

Data analysis was performed using STATA/SE for Windows, version 14 (StataCorp, college Station, TX, U.S.A.). Statistical significance was defined as p < 0.05.

### Results and discussion

Six hundred ninety-two patients were enrolled, 61% of whom were male, with a mean age of 65.4 ±16.9 years. Table 1 shows the mean characteristics of all patients and of the derivation and validation cohorts. AKI occurrence within 7 days was 38.7% in the derivation cohort and 36.3% in the validation cohort. Table 2 shows how AKI stages were distributed in the two cohorts. There was no significant difference between the two groups (p>0.05).

**Table 1:** Characteristics of patients, derivation and validation cohorts. There was no significant difference between the two groups (p>0.05).

Variables	All patient (n = 692)	Derivation cohort (n = 455)	Validation cohort (n = 237)
**Male, n (%)**	421(60.8)	279 (61.3)	142 (59.9%)
**Age (years), mean±SD**	65.4 ±16.9	65.9±16.8	64.4±17.0
**Weight (kg), median (IQR)**	75.0 (65.0–85.0)	75.0 (65.0–85.0)	71.5 (65.0–85.0)
**Height (cm), median (IQR)**	170 (165.-175.0)	170 (165.-175.0)	170.0 (168.0–177.0)
**Obese, n (%)**	111(16.0)	68 (14.9)	43 (18.1)
**Hypertension, n (%)**	352 (51.2)	233 (51.5)	119 (50.4)
**Diabetes mellitus type2, n (%)**	123 (17.9)	81 (17.8)	42 (17.9)
**eGFR ≥ 90 (ml/min/1.73 mq), n (%)**	428 (61.8)	282 (62.0)	146 (61.6)
**SOFA cv ≥2, n (%)**	276 (39.9)	191 (42.0)	85 (35.9)
**Lactate ≥2 (mmol/L), n (%)**	309 (44.7)	212 (46.6)	97 (40.9)
**PEEP (cmH2O), median(IQR)**	7.0 (5.0–8.0)	7.0 (5.0–8.0)	7 (5.0–8.0)
**PaCO2 (mmHg), median(IQR)**	39.0 (34.0–46.0)	39.0 (34.0–46.0)	38.4 (33.0–46.1)
**Surgery, n (%)**	282 (40.9)	185 (40.7)	97 (41.1%)
**AKI presence within 7 days, n (%)**	262 (37.9)	176 (38.7)	86 (36.3)
**(TIMP-2)•(IGFBP7) ((ng/ml)2/1000) ≥0.3, n (%)**	370 (53.5)	246 (54.1)	124 (52.3)
**Nephrotoxic drugs**^a^**, n (%)**	140 (20.3)	102 (22.5)	38 (16.1%)

^a^Nephrotoxic drugs included aminoglycosides, non-steroidal anti-inflammatory drugs, and vancomycin.

eGFR = estimated glomerular filtration rate; PEEP = positive end-expiratory pressure; PaCO2 = partial pressure of carbon dioxide in arterial blood SOFAcv = cardiovascular sequential organ failure assessment; TIMP-2, tissue inhibitor of metalloproteinases 2.; IGFBP7, insulin-like growth factor-binding protein 7

**Table 2:** Distribution of the AKI stages in the derivation and validation cohorts. There was no significant difference between the two groups (p>0.05).

AKI occurrence patients, n (%)	All patient 262 (37.9)	Derivation cohort 176 (38.7)	Validation cohort 86 (36.3)
**Stage 1**	158 (60.3)	105 (23.1)	53 (22.4)
**Stage 2**	66 (25.2)	45 (9.9)	21 (8.9)
**Stage 3**	38 (14.5)	26 (5.7)	12 (5.1)

Obesity (Body Mass Index (BMI > 30), admission eGFR, cardiovascular SOFA (SOFAcv), Lactate and (TIMP-2)•(IGFBP7) were significantly associated with AKI in a multivariate logistic regression (Table 3). More specifically, these variables were associated with an increased risk of AKI: eGFR< 90 ml/min /1.73 mq (OR 2.78; 95% CI 1.78–4.35; p<0.001); SOFAcv ≥ 2 (OR 2.23; 95% CI 1.48–3.37; p<0.001); lactate ≥ 2 mmol/L (OR 1.81; 95% CI 1.19–2.74; p = 0.005) and (TIMP-2)•(IGFBP7) ≥0.3 (OR 1.65; 95% CI 1.08–2.52; p = 0.019). In this model, the area under the ROC curve was 0.73 (95%CI 0.68–0.78); goodness of fit was p = 0.5232; Se = 57.4 and Sp = 80, IDI 0.012 p = 0.0139. We converted the OR into integer single risk scores. By summing the component variables, the total score can range from a minimum of 0 to a maximum of 10 points (Table 4).

**Table 3:** Predictors of AKI in derivation cohorts (n = 455).

	OR	95% CI		p-value
		Lower	Upper	
**Obese vs non-obese**	1.28	0.72	2.26	0.399
**eGFR < 90 (ml/min/1.73 mq)**	2.78	1.78	4.35	<0.001
**SOFA cv ≥2**	2.23	1.48	3.37	<0.001
**Lactate ≥2 (mmol/L)**	1.81	1.19	2.74	0.005
**(TIMP-2)•(IGFBP7) ≥ 0.3 ((ng/ml)2/1000)**	1.65	1.08	2.52	0.019

eGFR = estimated glomerular filtration rate SOFAcv = cardiovascular sequential organ failure assessment; TIMP-2, tissue inhibitor of metalloproteinases 2.; IGFBP7, insulin-like growth factor-binding protein 7

**Table 4:** AKI risk score of the final model.

Risk factors	Points
**Obese vs non-obese**	1
**eGFR < 90 (ml/min/1.73 mq)**	3
**SOFA cv ≥2**	2
**Lactate ≥2(mmol/L)**	2
**(TIMP-2)•(IGFBP7) ≥0.3 ((ng/ml)2/1000)**	2

eGFR = estimated glomerular filtration rate SOFAcv = cardiovascular sequential organ failure assessment; TIMP-2, tissue inhibitor of metalloproteinases 2.; IGFBP7, insulin-like growth factor-binding protein 7

After that, we performed the final logistic regression model on the validation cohort.

In this “validation” model, the area under the ROC curve was 0.76 (95%CI 0.69–0.82); goodness of fit was p = 0.3204; Se = 48.8 and Sp = 88.7. Finally, through a sensitivity analysis and medical considerations, we defined a low-risk of developing AKI as a score below the cut-off value 5.

Regarding the AKI Risk Score, both cohorts showed similar results: in the derivation cohort, 44% of patients had a Risk Score of less than 5 points compared to 45% in the validation cohort.

**Fig 1:**
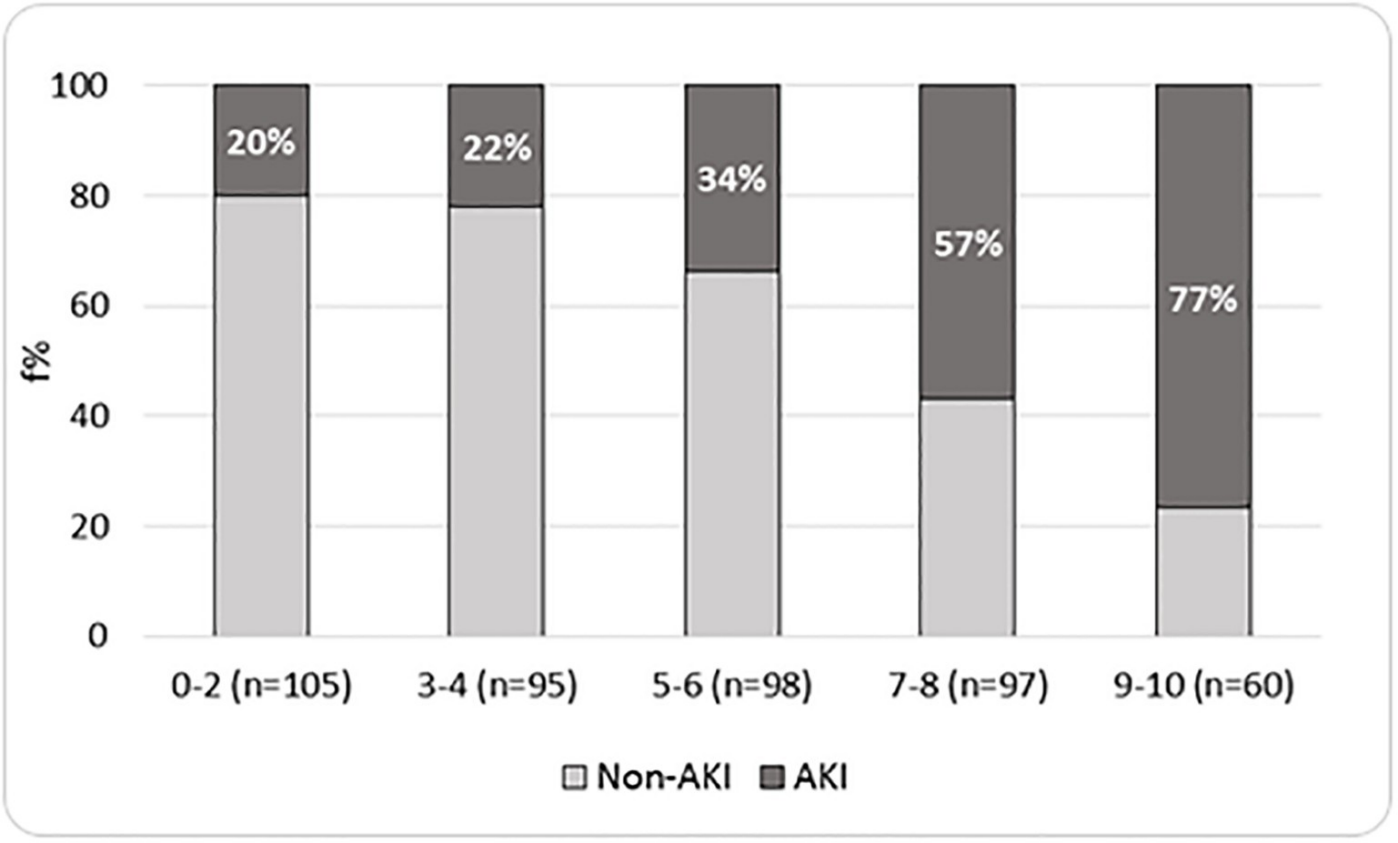
Distribution of AKI by AKI risk score levels—Derivation cohort (n = 455).

**Fig 2:**
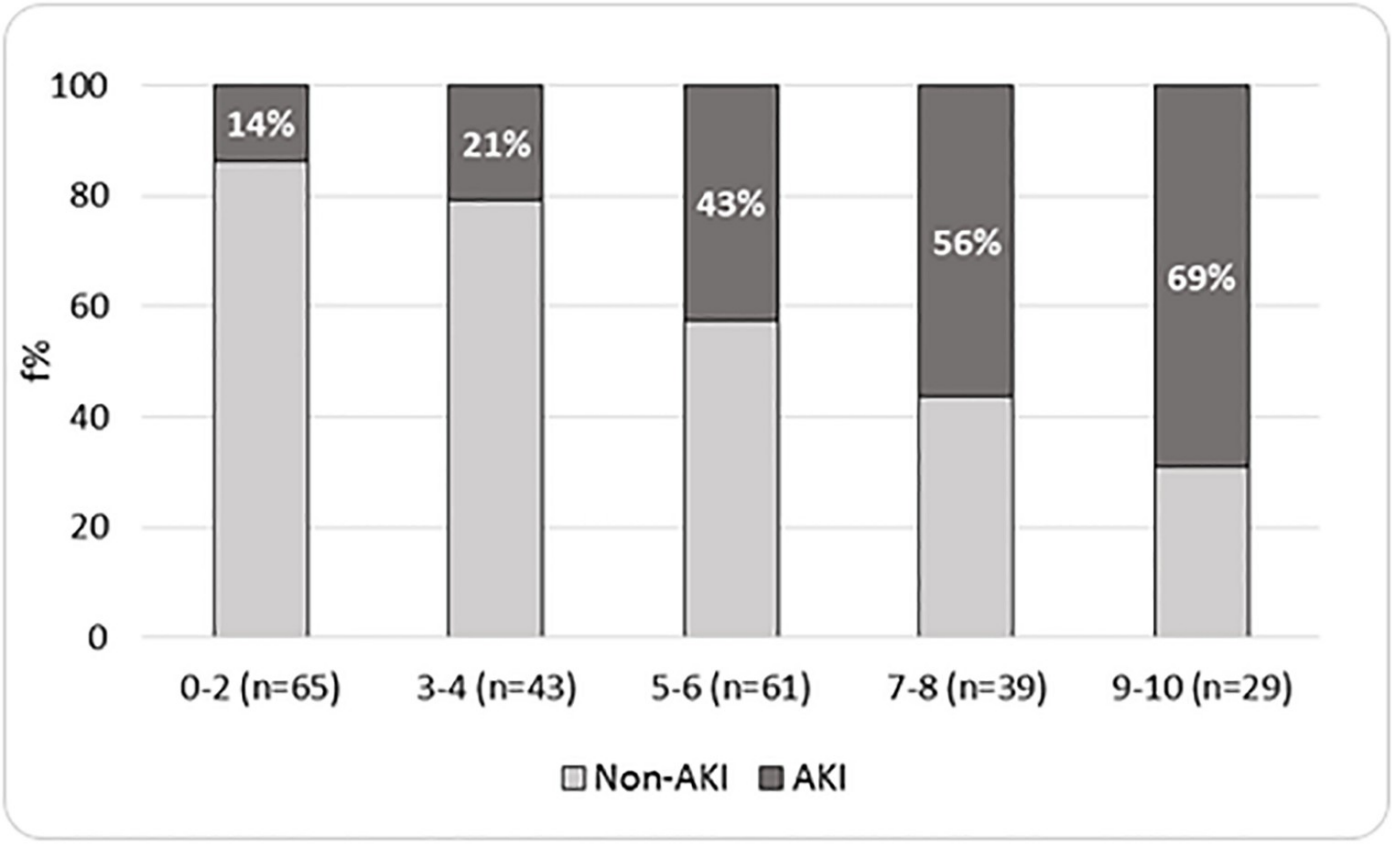
Distribution of AKI by AKI risk score levels—Validation cohort (n = 237).

For an easy-to-use AKI risk score, we stratified patients into different AKI Risk Score levels: 0–2; 3–4; 5–6; 7–8 and 9–10 (Figs 1 and 2). In both cohorts, as shown in Figs 1 and 2, we observed that the proportion of AKI patients was higher in the higher score levels. More precisely, in the derivation and validation cohorts, AKI was present in, respectively, 20% and 14% of participants belonging to the lowest risk group, 22% and 21% to the 3–4 risk level group, 34% and 43% to the 5–6 risk level group, 57% and 56% to the 7–8 risk level group, and 77% and 69% to the highest risk level group. In both cohorts, the risk of developing AKI was proportional to the increase in the level of risk (p for trend <0.0001).

In the past 25 years, the incidence of AKI has increased by at least 20 times [16]. Recently, Bellomo et al. and Palevsky et. al. have shown that the mortality rate of critically ill patients with AKI was 40%~70% [17,18]. AKI is not only a medical problem but has also become a major public health concern.

The Annual AKI-associated costs represent a substantial component of the National Health System budget, even when the patients recover renal function. In fact, AKI diagnosis is associated with a length of stay 2.57 times higher than that for admission without AKI; and 59.89% of the critical care bed days are for people with AKI [19]. Pannu et al. found that, among the patients who had developed severe AKI (KDIGO stage 2 or 3), 30.8% died and 2.1% progressed to CKD stage 5, requiring dialysis within a 34-month period [20].

Therefore, early recognition of AKI is relevant for critical care physicians to improve the quality of interventions in order to avoid or limit the progression of renal disease.

Since the treatment is largely supportive, AKI prevention becomes mandatory in critically ill patients [21].

Our study has established a new simple prediction score to quickly predict AKI at any stage up to 7 days.

Previously, we demonstrated that the development of AKI at any stage during the first week of ICU stay can be quickly predicted based on clinical information and (TIMP-2)•(IGFBP7) urine measurement collected up to 24 hours after ICU admission (*Ferrari F*, *submitted to Scientific Reports*, *2018*). To speed up the risk evaluation, in this study we considered variables available by one hour.

The final model included eGFR< 90 ml/min /1.73 mq; SOFAcv ≥ 2; lactate ≥ 2 mmol/L and (TIMP-2)•(IGFBP7) ≥ 0.3 ((ng/ml)2/1000).

While eGFR gives information on renal function at admission, including anthropometric characteristics, SOFAcv and lactate levels mirror a modified perfusion that might affect renal autoregulation.

Recently, perfusion pressure has been considered as an indicator for the prevention of AKI. In terms of perfusion pressure, diastolic perfusion pressure (DPP) and mean perfusion pressure (MPP) should be pointed out [22]. Two observational studies revealed that lower DAP or decreased MPP were associated with septic AKI [23,24].

Therefore, since decreased DAP is associated with AKI, catecholamines may be effective in preventing AKI [25].

Instead, obesity is associated with an increased risk for AKI in critically ill patients admitted to a medical or surgical ICU [26] after cardiac [27,28] and bariatric surgery [29].

Obesity leads to both venous congestion and poor arterial organ perfusion: in effect, obese patients show an impaired diastolic function due to left ventricular hypertrophy and adipocytes infiltration of the myocardium [30] and an elevated intra-abdominal pressure, which needs a higher Positive End Expiratory Pressure (PEEP) in mechanically ventilated patients [31, 32].

Furthermore, obesity impacts many pharmacokinetic factors: the weight-based dose of hydrophilic drugs might reach the nephrotoxic threshold [33]; on the other hand, adipocytes are involved in the secretion of inflammatory mediators that can lead to kidney damage [34].

Contrasting results regarding interventions to prevent AKI have led to disappointment regarding the use of biomarkers alone [35,36]. Nevertheless, (TIMP-2)•(IGFBP7) improved the predictive performance at ICU admission of a clinical model for AKI at any stage (*Ferrari F*, *submitted to Scientific Reports 2018*, [37], even if its best performance is to identify patients at risk of developing severe (stage 2–3) AKI [38–40].

Including a biomarker admission measurement improves the predictive ability of the logistic model and allows for speedier diagnoses, even if glomerular filtration has not yet decreased [13].

The strength of our score is that it quickly identifies patients with a high-risk of developing AKI at any stage through simple and quick information available at ICU admission. Compared to previous studies [38–40], a combined approach could improve the diagnostic prediction of the biomarker alone to identify less severe AKI as well.

However, our study still has some limitations: it was a retrospective study which limits the generalization of its findings and a single-centre study which may not be directly applicable to other patient populations. Furthermore, (TIMP-2)•(IGFBP7) was measured only at ICU admission. There are no studies that evaluate whether serial measurements might improve predictive performance.

## Conclusion

We have shown that AKI development within the first week of an ICU stay, as defined by the KDIGO criteria [7], might be identified from a prediction model that uses data routinely available one hour after admission.

Furthermore, (TIMP-2)•(IGFBP7) improves the prediction ability of the model and might allow for speedier diagnoses, even if glomerular filtration has not yet decreased.

## Supporting information

S1 Fig Flow chart of study population selection and research process. (TIF)

S1 File Information about the patient records. (DOCX)

S2 File Database.csv: Anonymized dataset of the study. (CSV)
