# Supplementary figures and images for: Development and validation of quick Acute Kidney Injury-score (q-AKI) to predict acute kidney injury at admission to a multidisciplinary intensive care unit
Source: PLoS One. 2019 Jun 20;14(6):e0217424. doi: 10.1371/journal.pone.0217424 (PMC6586286; doi:10.1371/journal.pone.0217424)

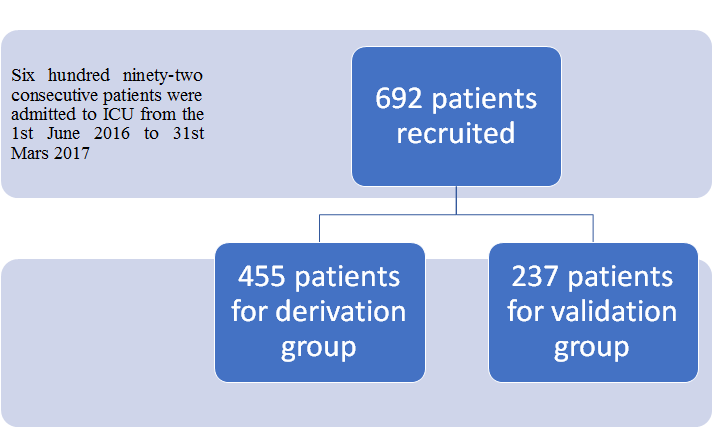

Supplement: S1 Fig — (TIF) [file pone.0217424.s001.tif]
