# Supplementary material for: Development and validation of quick Acute Kidney Injury-score (q-AKI) to predict acute kidney injury at admission to a multidisciplinary intensive care unit
Source: PLoS One. 2019 Jun 20;14(6):e0217424. doi: 10.1371/journal.pone.0217424 (PMC6586286; doi:10.1371/journal.pone.0217424)
Supplement: S1 File — (DOCX) [file pone.0217424.s002.docx]

**Information about the patient records**

All collected data will be identified and stored in such a way as to protect patient confidentiality. The

staff involved in the collection and management of data will not use or disseminate such information

for purposes other than those provided for the realization of the study.

All data will be collected and processed in a completely anonymous way, in compliance with the

guarantee terms of privacy and as approved by the Ethics Committee of San Bortolo Hospital in

Vicenza, and stored at the International Renal Research Institute Vicenza, as required by current

regulations. The export of records for the further processing and analysis is anonymised.

For the retrospective study, we based on the Italian normative n. 9/2014 (“*in merito al trattamento dei dati personali effettuato per scopi di ricerca scientifica dell’11 dicembre 2014*”), which let use to aggregate data without a written informed consent.
